# Myosteatosis Differentially Affects the Prognosis of Non-Metastatic Colon and Rectal Cancer Patients: An Exploratory Study

**DOI:** 10.3389/fonc.2021.762444

**Published:** 2021-11-11

**Authors:** Lara Pozzuto, Marina Nogueira Silveira, Maria Carolina Santos Mendes, Lígia Traldi Macedo, Felipe Osório Costa, Carlos Augusto Real Martinez, Cláudio Saddy Rodrigues Coy, Ademar Dantas da Cunha Júnior, José Barreto Campello Carvalheira

**Affiliations:** ^1^ Division of Oncology, Department of Anesthesiology, Oncology and Radiology, School of Medical Sciences, State University of Campinas (UNICAMP), Campinas, Brazil; ^2^ Division of Gatrointestinal Surgery, Department of Surgery, School of Medical Sciences, State University of Campinas (UNICAMP), Campinas, Brazil; ^3^ Hematology and Oncology Clinics, Cancer Hospital of Cascavel, União Oeste de Estudos e Combate ao Câncer (UOPECCAN), Cascavel, Brazil; ^4^ Department of Internal Medicine, State University of Western Paraná (UNIOESTE), Cascavel, Brazil

**Keywords:** skeletal muscle radiodensity, cancer, survival, computerized tomography, sarcopenia, skeletal muscle mass

## Abstract

Body composition performed by computed tomography (CT) impacts on cancer patients’ prognoses and responses to treatment. Myosteatosis has been related to overall survival (OS) and disease-specific survival in colorectal cancer (CRC); however, the independent impact of the association of myosteatosis with prognosis in colon cancer (CC) and rectal cancer (RC) is still unclear. CT was performed at the L3 level to assess body composition features in 227 patients with CRC. Clinical parameters were collected. Overall survival (OS) was the primary outcome, and the secondary outcome was disease-free survival (DFS). Skeletal muscle attenuation and intramuscular adipose tissue area were associated with DFS (p = 0.003 and p = 0.011, respectively) and OS (p < 0.001 and p < 0.001, respectively) in CC patients but not in RC patients. Only the skeletal muscle area was associated with better prognosis related to OS in RC patients (p = 0.009). When CC and RC were analyzed separately, myosteatosis influenced survival negatively in CC patients, worsening DFS survival (hazard ratio [HR], 2.70; 95% confidence interval [CI], 1.07–6.82; p = 0.035) and OS (HR, 5.76; 95% CI, 1.31–25.40; p = 0.021). By contrast, the presence of myosteatosis did not influence DFS (HR, 1.02; 95% CI, 0.52–2.03; p = 0.944) or OS (HR, 0.76; 95% CI, 0.33–1.77; p = 0.529) in RC patients. Our study revealed the interference of myosteatosis in the therapy and survival of patients with CC but not in those with RC, strengthening the value of grouping the two types of cancer in body composition analyses.

## Introduction

Cancer is a condition that affects millions of people and is among the leading causes of death worldwide, with increasing number of cases each year. In 2020, the estimated number of new cases was 19.3 million, with a forecast of up to 30.2 million by 2040 (1). Colorectal cancer (CRC) is among the most incident types of the disease, occupying the third position of highest incidence in both sexes (1, 2).

Body composition impacts cancer patients’ prognosis, the response to treatment, and consequently, the survival of these individuals (3–5). Computed tomography (CT) is an effective and accurate method for identifying body features that may interfere with patient treatment and prognosis (3); indeed, CT scanning is the most suitable method for assessing body composition in cancer patients (6) to predict toxicity, tolerance to treatment, and survival (7, 8). One of the body composition features that can be identified through CT scan is loss of muscle tissue, which can be caused by reduced muscle fiber number and diameter as well as by fat infiltration and collagen deposit into the muscle (9, 10). Intramuscular fat invasion is known as myosteatosis and determines low muscle radiodensity on CT scan (4, 11); this feature can occur in patients with different body mass indexes (12). Importantly, myosteatosis is strongly related to shorter survival in certain cancer patients (5, 13, 14).

Relevant to oncological patients’ survival outcomes, myosteatosis in CRC is also related to patients’ disease-free survival (DFS) and overall survival (OS) (15–18); studies showed that this disorder is related to patients’ postoperative results and treatment (19–22). Notably, McSorley et al. identified that among the parameters of body composition by CT—sarcopenia, myosteatosis, and visceral obesity—only myosteatosis was associated with OS and disease-specific survival, but not independently of inflammatory parameters (15), indicating that the effects of covariables may strongly impact the influence of myosteatosis on survival outcomes. Along these lines, some studies do not take into consideration the cancer clinical stage (CS), generalizing the findings to varying times of the diseases and outcomes (23–25). Furthermore, body composition studies in non-metastatic CRC rarely distinguish colon cancer (CC) and rectal cancer (RC) (15–17, 21, 26).

Although CC and RC have similar pathophysiology, the chemotherapy and radiotherapy approaches for these entities are strikingly different. Therefore, the present study aimed to assess the association of myosteatosis in DFS and OS of patients with non-metastatic CC and RC and how this body composition feature may influence the patients’ therapy.

## Material and Methods

### Study Population

This retrospective–observational cohort involved patients diagnosed with non-metastatic CRC between January 2010 and December 2017 at the University of Campinas (UNICAMP University Hospital). Information was gathered from electronic or physical medical records from the diagnosis period until the last day of follow-up or death. Inclusion criteria were as follows: histologically confirmed CRC adenocarcinoma; patients submitted to curative-intent surgery; clinical stages (CS) I to III according to the 8th AJCC cancer manual (27); abdominal CT scans performed 3 months before or after the diagnosis and available electronically in the picture archiving and communication system; and availability of key clinical, demographic, and anthropometric data of interest. Patients diagnosed with cancer other than adenocarcinoma or primary cancer at other concomitant sites, patients for whom only low-quality CT was available or contrast CT was unavailable, those in stage IV, those for whom treatment data were not reported, and those with CRC *in situ* were excluded. The local Institutional Review Board approved this study (CAAE number: 84469318.2.0000.5404), as principles recommended by the Declaration of Helsinki have been respected and obtained a waiver for the consent form.

### Body Composition Evaluation

Computed tomography images were evaluated to obtain the patients’ body composition. CT images were routinely performed for cancer staging, collected weight and height data, and calculated BMI. The images were analyzed using the software viewer Software SliceOMatic V.5.0 (TomoVision, Canada). The standard Hounsfield units (HU) settled for tissues were −29 to 150 for skeletal muscle (SM), −150 to −50 for visceral adipose tissue (VAT), and −190 to –30 for intramuscular adipose tissue (IMAT) and subcutaneous adipose tissue (SAT). Skeletal muscle groups evaluated include the psoas, abdominal, rectus abdominal, and paravertebral muscles (6, 28). Skeletal muscle and subcutaneous or visceral adipose tissue were measured in units of square centimeters (cm²) and normalized for height in square meters (m²) and reported as SM index (SMI), SAT index (SFI), and VAT index (VFI) in cm²/m² units. SM density was measured as the mean radiological tissue attenuation in HU (29, 30). Myosteatosis in patients was determined with cutoff points of <41 HU for patients with BMI ≤24.9 and <33 HU for those with BMI ≥25, according to Martin et al. (12). Two consecutive images of the third lumbar vertebra were acquired, and the image analyzes were performed by two independent evaluators (MS and LP), who were blind to the outcomes under study. Coefficients of variation for the cross-sectional areas analyzed were 1.07%, 1.05%, 1.61%, and 3.57% for SM and SAT, VAT, and IMAT, respectively, and 1.60% for SM density.

### Previously Established Prognostic Factors and Other Clinical Parameters

Data were retrospectively collected from medical records and entered into the electronic tool REDCap hosted at the University of Campinas (31). Clinical parameters collected included age, sex, BMI, weight loss (less than 5% or more than 5% of the original weight), alcoholism, smoking, Charlson Comorbidity Index, tumor stage, emergency surgery neoadjuvant and adjuvant therapy, biochemical tests, and toxicity data during treatment (according to NCI CTCAE version 5.0) (32).

### Endpoints

The primary outcome was overall survival (OS), calculated between the time of diagnosis, and death from any cause. The secondary outcome was disease-free survival (DFS), calculated between the time of diagnosis and disease progression or death from CRC. The date of death was determined from the death certificate’s date attached to the medical record or from information obtained by telephone contact with family members. Living patients were censored on the date of the last follow-up.

### Statistical Analysis

Categorical variables were analyzed using χ^2^ or Fisher’s exact test, when appropriate, and Student’s t-test for continuous variables, presented in the baseline table and the table with characteristics according to the patients’ chemotherapy treatment. Kaplan–Meier curves were created to evaluate the effect of myosteatosis on the survival of patients with CRC, CR, and CC, and the differences between the curves were assessed using log-rank tests. Obtaining hazard ratios (HR) and respective confidence intervals (CI), proportional risk models were performed using Cox regression with 95% CI for disease-free survival and overall survival. In the multivariate analysis, variables with p <0.1 identified in the univariate were used, and adjustments were made in Cox’s multivariate regression analysis for age, Charlson Comorbidity Index (CCI) (33), alcohol consumption, and AJCC stage. Kaplan–Meier curves were created to evaluate the effect of myosteatosis on the survival of patients with CRC, CR, and CC, and the differences between the curves were assessed using log-rank tests. Statistical significance was determined with a two-sided p-value <0.05, and the software used for the analyses was Stata, version 12.0 (StataCorp LP, College Station, United States).

## Results

### Baseline Clinical and Demographic Characteristics

**Figure 1:**
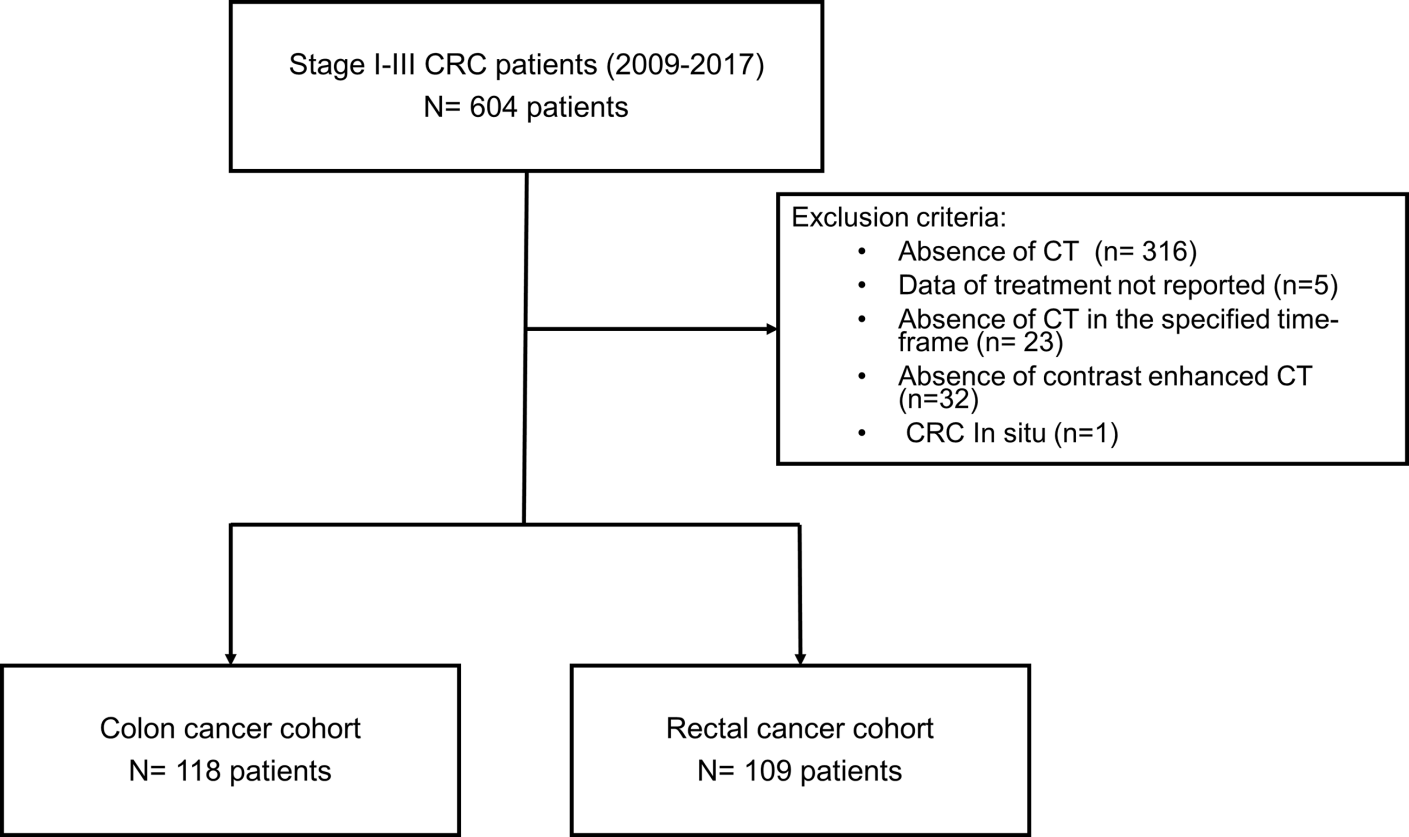
Flow chart showing the inclusion and exclusion process for patients treated for CRC stage I-III.

The study cohort included 227 CRC patients, 118 with colon cancer (CC), and 109 with rectal cancer (RC) (**Figure 1**). Six hundred and four patients were treated for stage I–III CRC, between 2009 and 2017, in our data collection. Of these, 288 patients had CT available for analysis. Patients with missing treatment information (n = 5), lacking CT either at the established time (n = 23) or without contrast (n = 32), and *in situ* CRC (n = 1) were excluded.

The clinical and demographic characteristics of the patients, according to the presence of myosteatosis, are described in **Table 1** for CC and RC patients. For CC, most patients had less than 65 years, while most significant RC patients with myosteatosis had more than 65 years. Still, myosteatosis was more prevalent in patients older than 65 years in both colon and rectal cancer. CC or RC patients with myosteatosis had the worst Charlson Comorbidity Index.

**Table 1:** Selected demographic and disease characteristics according to myosteatosis of stage I–III colon and rectal cancer patients.

Characteristic	Colon cancer			Rectal cancer		
	No myosteatosis, n = 32	Myosteatosis, n = 86	p value	No myosteatosis, n = 45	Myosteatosis, n = 64	p value
Age, № (%)						
Less than 65 years	29 (90.6)	53 (61.6)	**0.003**^a^	33 (73.3)	29 (45.3)	**0.004** ^b^
More than 65 years	3 (9.4)	33 (38.4)		12 (26.7)	35 (54.7)	
Sex, № (%)						
Female	16 (50.0)	46 (53.5)	0.736^b^	26 (57.8)	37 (57.8)	0.997^b^
Male	16 (50.0)	40 (46.5)		19 (42.2)	27 (42.2)	
BMI, median (IQR)	23.8 (19.6–25.5)	24.2 (20.9–27.7)	0.357^c^	25.8 (24.2–27.2)	25.7 (23.1–29.4)	0.781^c^
Weight loss, № (%)						
Less than 5%	9 (28.1)	18 (20.9)	0.281^b^	19 (42.2)	26 (40.6)	0.976^b^
More than 5%	20 (62.5)	67 (77.9)		26 (57.8)	36 (56.3)	
NR	3 (9.4)	1 (1.2)		0 (0.0)	2 (3.1)	
Alcohol consumption, № (%)						
No	21 (65.6)	58 (67.4)	0.858^b^	30 (66.7)	36 (56.3)	0.366^b^
Yes	9 (28.1)	27 (31.4)		15 (33.3)	26 (40.6)	
NR	2 (6.3)	1 (1.2)		0 (0.0)	2 (3.1)	
Smoking, № (%)						
No	19 (59.4)	47 (54.6)	0.564^b^	25 (55.6)	28 (43.7)	0.208^b^
Yes	12 (37.5)	38 (44.2)		19 (44.2)	35 (54.7)	
NR	1 (3.1)	1 (1.2)		1 (2.2)	1 (1.6)	
Charlson Comorbidity Index, № (%)						
2–3	26 (81,2)	27 (31.4)	**<0.001** ^a^	26 (57.8)	17 (26.6)	**0.001** ^a^
≥4	6 (18.8)	59 (68.6)		19 (42.2)	47 (73.4)	
Stage, № (%)						
I–II	10 (31.2)	34 (39.5)	0.408^b^	33 (73.3)	41 (64.1)	0.325^b^
III	22 (68.8)	52 (60.5)		11 (24.5)	21 (32.8)	
TXNXM0	0 (0.0)	0 (0.0)		1 (2.2)	2 (3.1)	
Emergency surgery, № (%)	10 (31.3)	27 (31.4)	0.988^b^	2 (4.4)	0 (0.0)	0.164^a^
Neoadjuvant treatment, № (%)	0 (0.0)	0 (0.0)		36 (80.0)	50 (78.1)	0.813^b^
Adjuvant treatment, № (%)	31 (96.9)	63 (73.3)	**0.004** ^a^	31 (68.9)	44 (68.8)	0.988^b^

BMI, body mass index, IQR, interquartile range; SAT, subcutaneous adipose tissue; SD, standard deviation.

a Fisher’s exact test.

b Chi-square test.

c Student’s t test. Bold was used to highlight values that were statistically significant (< 0.05).

Emergency surgery was performed on 39 (17%) patients, 86 (38%) had neoadjuvant therapy, and 169 (74%) had adjuvant treatment. Interestingly, 97% of patients with CC without myosteatosis were able to undergo adjuvant treatment, while significantly fewer patients undergo adjuvant therapy in the presence of myosteatosis (**Table 1**).

Among CC patients, just men with myosteatosis showed significant reduction in skeletal muscle area (p = 0.020) and SMI (p = 0.037). On the other hand, all patients with myosteatosis showed significantly larger IMAT area (p < 0.001). Regarding inflammatory parameters, myosteatosis was associated with lower LMR indices (p = 0.013) and higher PLR indices (p = 0.003) (**Supplementary Table 1**). As for patients with rectal cancer, in contrast, in both males and females, myosteatosis was related to minor skeletal muscle and SMI areas and larger IMAT areas. Besides, myosteatosis was related to higher VAT and VATI areas and SAT attenuation (**Supplementary Table 2**).

### Association of Body Composition With Survival Outcomes

**Figure 2:**
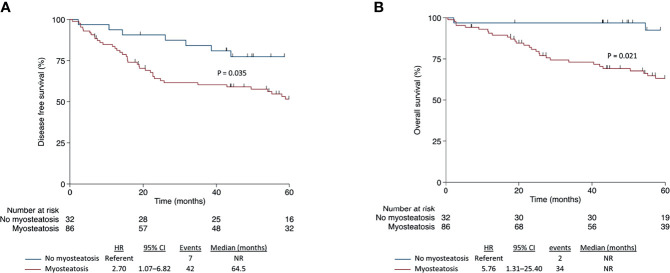
Disease-free survival **(A)** and overall survival **(B)** according to myosteatosis in patients with colon cancer.

**Figure 3:**
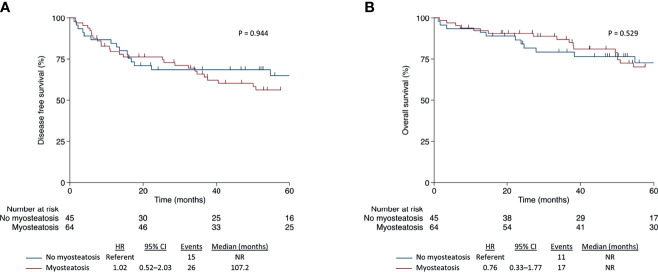
Disease-free survival **(A)** and overall survival **(B)** according to myosteatosis in patients with rectal cancer.

Body composition features associate differently in CC or RC patients. Skeletal muscle attenuation and intramuscular adipose tissue area were associated with disease-free survival and overall survival in CC patients, but not with RC patients. Only SM areas were associated with better prognosis related to OS in RC patients (**Tables 2** and **3**). Kaplan–Meier curves show that myosteatosis influences survival negatively in CC patients, worsening disease-free survival (hazard ratio [HR], 2.70; 95% confidence interval [CI], 1.07–6.82; p = 0.035) and overall survival (HR, 5.76; 95% CI, 1.31–25.40; p = 0.021) (**Figures 2A, B**). In opposite, the presence of myosteatosis did not influence DFS (HR, 1.02; 95% CI, 0.52–2.03; p = 0.944) or OS (HR, 0.76; 95% CI, 0.33–1.77; p = 0.529) in RC patients (**Figures 3A, B**).

**Table 2:** Univariate and multivariate COX regression analyses of body composition features of colon cancer patients.

	Disease-free survival						Overall survival					
Characteristic	Univariate analysis			Multivariate analysis			Univariate analysis			Multivariate analysis		
	HR	95% CI	*p* value	HR	95% CI	*p* value	HR	95% CI	*p* value	HR	95% CI	*p* value
Skeletal muscle												
Area (cm^2^)	0.99	0.98–1.00	0.170				0.99	0.98–1.00	0.066			
SMI (cm^2^/m^2^)	0.97	0.95–1.00	0.077				0.96	0.93–1.00	**0.045**	0.96	0.93-1.00	0.051
Attenuation (HU)	0.95	0.92–0.98	**0.001**	0.95	0.91–0.98	**0.003**	0.91	0.87–0.95	**<0.001**	0.91	0.87-0.95	**<0.001**
IMAT, area (cm^2^)	1.03	1.00–1.05	**0.024**	1.03	1.01–1.06	**0.011**	1.05	1.02–1.07	**<0.001**	1.06	1.03-1.09	**<0.001**
Visceral adipose tissue												
VAT, area (cm^2^)	1.00	1.00–1.00	0.767				1.00	1.00–1.01	0.567			
VATI (cm^2^/m^2^)	1.00	0.99–1.01	0.928				1.00	0.99–1.01	0.612			
VAT attenuation (HU)	1.01	0.98–1.03	0.500				1.01	0.98–1.04	0.383			
Subcutaneous adipose tissue												
SAT, area (cm^2^)	1.00	1.00–1.00	0.899				1.00	1.00–1.00	0.938			
SATI (cm^2^/m^2^)	1.00	0.99–1.01	0.856				1.00	0.99–1.01	0.996			
SAT attenuation (HU)	1.00	0.99–1.01	0.966				1.00	0.99–1.01	0.868			

The Cox model was adjusted for age (categorical), Charlson Comorbidity Index (categorical), alcohol consumption (categorical), and AJCC stage (categorical).

CI, confidence interval; HR, hazard ratio; IMAT, intramuscular adipose tissue; SAT, subcutaneous adipose tissue; SATI, subcutaneous fat index; SMI, skeletal muscle index; VAT, visceral adipose tissue; VATI, visceral fat index. Bold was used to highlight values that were statistically significant (< 0.05).

**Table 3:** Univariate and multivariate COX regression analyses of body composition features of rectal cancer patients.

	Disease-free survival						Overall survival					
Characteristic	Univariate analysis			Multivariate analysis			Univariate analysis			Multivariate analysis		
	HR	95% CI	*p* value	HR	95% CI	*p* value	HR	95% CI	*p* value	HR	95% CI	*p* value
Skeletal muscle												
Area (cm^2^)	0.99	0.98–1.00	0.150				0.98	0.97–1.00	**0.009**	0.98	0.96-0.99	**0.009**
SMI (cm^2^/m^2^)	0.97	0.94–1.01	0.130				0.95	0.91–0.99	**0.022**	0.96	0.91-1.00	0.061
Attenuation (HU)	0.98	0.95–1.02	0.307				0.98	0.95–1.02	0.435			
IMAT, area (cm^2^)	1.01	0.98–1.04	0.542				1.00	0.96–1.05	0.867			
Visceral adipose tissue												
VAT, area (cm^2^)	1.00	0.99–1.00	0.439				1.00	0.99–1.00	0.272			
VATI (cm^2^/m^2^)	1.00	0.99–1.01	0.588				1.00	0.98–1.01	0.501			
VAT attenuation (HU)	1.03	1.00–1.06	**0.047**	1.02	0.99–1.05	0.196	1.04	1.00–1.07	**0.030**	1.03	0.99-1.06	0.123
Subcutaneous adipose tissue												
SAT, area (cm^2^)	1.00	0.99–1.00	0.350				1.00	0.99–1.00	0.467			
SATI (cm^2^/m^2^)	1.00	0.99–1.00	0.408				1.00	0.99–1.01	0.717			
SAT attenuation (HU)	1.02	1.00–1.04	0.077				1.02	1.00–1.04	0.098			

The Cox model was adjusted for age (categorical), Charlson Comorbidity Index (categorical), alcohol consumption (categorical), and AJCC stage (categorical).

CI, confidence interval; HR, hazard ratio; IMAT, intramuscular adipose tissue; SAT, subcutaneous adipose tissue; SATI, subcutaneous fat index; SMI, skeletal muscle index; VAT, visceral adipose tissue; VATI, visceral fat index. Bold was used to highlight values that were statistically significant (< 0.05).

### Association Between Adjuvant and Neoadjuvant Treatment Characteristics According to Myosteatosis

Interestingly, we identified that myosteatosis in patients with CC was significantly related to the type of chemotherapy and adjuvant treatment duration. Specifically, fewer patients with CC and myosteatosis were exposed to oxaliplatin, and 55.6% of these patients completed adjuvant regimen, while 80.7% of CC patients without myosteatosis received the preplanned adjuvant treatment (p = 0.031). The presence of myosteatosis affected chemotherapy tolerance predominantly by increasing toxicity-related treatment interruption (p = 0.005). In RC, on the other hand, no statistically significant difference was found between myosteatosis and treatment tolerance, highlighting the differences between patients with CC and those with RC (**Table 4**).

**Table 4:** Selected adjuvant and neoadjuvant treatment characteristics according to myosteatosis of stage I–III colon and rectal cancer patients, respectively.

Characteristic	Colon cancer patients that underwent adjuvant treatment		
	No myosteatosis, n = 31	Myosteatosis, n = 63	p value
Chemotherapy regimen			
5-FU plus oxaliplatin	23 (82.1)	36 (57.1)	**0.031**
5-FU plus leucovorin	5 (17.9)	27 (42.9)	
Adjuvant treatment complete, № (%)	25 (80.7)	35 (55.6)	**0.022** ^a^
Adjuvant treatment interrupted, № (%)			
Toxicity	1 (3.2)	17 (27.0)	**0.005** ^a^
Performance deterioration	1 (3.3)	2 (3.2)	1.000^a^
Progression	1 (3.2)	4 (6.4)	1.000^a^
Abandonment	2 (6.3)	4 (6.4)	1.000^a^
NR	1 (3.2)	1 (1.6)	1.000^a^
Toxicity grades III–IV, № (%)			
All	12 (26.1)	19 (39.6)	0.164^a^
Diarrhea	6 (19.4)	18 (28.6)	0.452^a^
Emesis	2 (6.5)	0 (0.0)	0.116^a^
Mucositis	3 (9.7)	2 (3.2)	0.327^a^
Hematological	1 (3.2)	11 (17.5)	0.096^a^
**Characteristic**	**Rectal cancer patients that underwent neoadjuvant treatment**		
	**No myosteatosis, n = 36**	**Myosteatosis, n = 50**	**p value**
5-FU or capecitabine plus radiotherapy, № (%)	36 (100.0)	50 (100.0)	1.00
Neoadjuvant treatment complete, № (%)	31 (86.1)	48 (96.0)	0.124^b^
Neoadjuvant treatment interrupted	5 (13.9)	2 (4.0)	
Toxicity grades III–IV, № (%)			
All	10 (27.8)	16 (32.0)	0.674^a^
Diarrhea	6 (16.7)	12 (24.0)	0.592^a^
Emesis	2 (5.6)	1 (2.0)	0.569^a^
Mucositis	0 (0.0)	2 (4.0)	0.508^a^
Hematological	2 (5.6)	0 (0.0)	0.172^a^

NR, not reported

a Fisher’s exact test.

b Chi-square test.

## Discussion

Myosteatosis in CC is associated with worse DFS and OS even after adjustments for age, CCI, alcoholism, and CS. In opposite, we did not detect that myosteatosis affected the survival outcomes in the entire population (CRC) or in RC alone. In accordance, myosteatosis prevented the completion rate of adjuvant chemotherapy in CC patients. On the other hand, we did not identify an association between myosteatosis and the completion rate of neoadjuvant systemic chemotherapy (NSC) to RC patients.

Myosteatosis is a factor that negatively affects survival (14, 34–36), and it is associated with increased chemotherapy toxicity (36–38) as well as with increased hospital readmissions due to postoperative complications (13, 22). Our findings extend these data by showing that myosteatosis prevented patients with CC from receiving the entire preplanned adjuvant chemotherapy regimen. In contrast, myosteatosis did not influence the NSC administration in RC patients. Interestingly, in a recent Latin-American-based study with non-metastatic CRC, body composition was not associated with survival outcomes (26), which corroborates the present results in the entire cohort. In aggregate, these data suggest that myosteatosis is a marker of postoperative frailty, which detects patients who performed worse during surgery and thus were unable to complete or even start adjuvant therapy, therefore jeopardizing the opportunity to offer patients a more satisfying quality of life and more prolonged survival.

Although CC and RC have different characteristics about the clinical management, treatment, and outcomes of each disease (25, 39, 40), our study is the first to systematically investigate the effect of myosteatosis on CC and RC separately. Saliently, for patients with stage III CC, adjuvant chemotherapy with fluoropyrimidine combined with oxaliplatin diminishes the risk of relapse and mortality, with a therapy duration that might be abbreviated to 3 months as effective as 6 months, particularly in the lower-risk subgroup and in specific conditions according to limit toxicities, such as sensitive neuropathy or thrombocytopenia (41). Recognizing the visible cost and disability to patients results in surgical complications; current investigations have concentrated on distinguishing modifiable biomarkers to advance perioperative risk stratification and purpose supportive management. We identified that myosteatosis in patients with CC was significantly related to the type of chemotherapy and adjuvant treatment duration. Moreover, the presence of myosteatosis affected chemotherapy tolerance predominantly by increasing toxicity-related treatment interruption. Therefore, the present data suggest that myosteatosis could be a biomarker associated with toxicity, which might be assessed previously to chemotherapy protocol in CC patients. In RC, in contrast, no statistically significant difference was observed between myosteatosis and treatment tolerance, indicating the treatment approach diversity between patients with CC and RC.

The reasons for the different impacts of myosteatosis on CC, RC DFS, and OS are not entirely clear. However, it is essential to observe that the differences between the two tissues begin in their embryonic origin, which generates differences in the local blood flow supply, different metabolic pathways, and consequently differences in tumor development (42). Colon and rectal cancers have different molecular patterns and differentiation profiles; tumor size, malignancy, and the T extension of the invasion are distinct (43–51). Such differences impact cancer treatment, therapy choice, response to therapy, and survival. While neoadjuvant chemotherapy’s efficacy is still under investigation for colon cancer (52), its use has been well established to treat rectal cancer and our negative findings might underline the importance of myosteatosis as a postoperative biomarker for CC using adjuvant chemotherapy. NSC approaches are considered standard of care in numerous other gastrointestinal tumor types such as gastric, esophageal, and pancreatic cancer (53–55), also in RC (56). Interestingly, in a previous study we also did not find a shorter survival in locally advanced esophageal cancer patients with myosteatosis that were not submitted to surgery, reinforcing the idea of myosteatosis as a marker of postoperative frailty (57). Moreover, the benefit of neoadjuvant chemotherapy regimens may be related to increased completion rate of subsequent treatments (58); thus, further studies evaluating myosteatosis as a marker for anticipating the use of chemotherapy in neoadjuvant, instead of in adjuvant setting, is warranted.

In the context of metastatic CRC (mCRC), low muscularity was associated with shorter DFS and OS in most studies (18, 59). However, some studies have not found an effect of sarcopenia at diagnosis on mCRC prognosis, despite the detection of a negative influence in OS caused by muscle mass loss during the chemotherapy period (60, 61). Regarding myosteatosis, the results are also controversial. Myosteatosis is associated with a worse prognosis in patients with mCRC in certain studies (18, 59); others do not find an association (60). Notably, we did not notice any study that assessed CC and CR separately in the metastatic context.

Our study presents key strengths, with a rigorous sample as to the selection and analysis of the CRC. We recognize some limitations in our study which are the retrospective design, the sample number, and the possibility of sealing bias due to the loss of cases due to the lack of CT. Furthermore, we did not have data on dietary intake, physical activity, socioeconomic status, and perioperative care support, which could reasonably have affected SMI, SMD, and outcomes. Therefore, further prospective studies are needed to confirm our findings.

## Conclusion

Our study clearly showed the interference of myosteatosis in the treatment and survival of patients with CC, but not in RC, reinforcing the importance of separating the two types of cancer in body composition studies. In addition, myosteatosis in the postsurgical recovery negatively affected for non-indication of adjuvant therapy and contributed to the striking difference we found between CC and CR.

## Data Availability Statement

The original contributions presented in the study are included in the article/**Supplementary Material**. Further inquiries can be directed to the corresponding author.

## Ethics Statement

The studies involving human participants were reviewed and approved by Comitê de Ética em Pesquisa (CEP) da Universidade Estadual de Campinas. Written informed consent for participation was not required for this study in accordance with the national legislation and the institutional requirements.

## Author Contributions

Conceptualization, LP, MS, MM, and JC. Data curation, MS, MM, and JC. Formal analysis, LP, MS, MM, and JC. Funding acquisition, JC. Methodology, LP, MS, and MM. Project administration, JC. Resources, JC. Supervision, MM and JC. Visualization, MS, LM, FC, CM, CC, and AC. Writing—original draft, LP, MS, MM, FC, and AC. Writing—review and editing, LP, MS, MM, LM, CM, CC, AC, and JC. All authors contributed to the article and approved the submitted version.

## Funding

This research was funded by Fundação de Amparo à Pesquisa do Estado de São Paulo (FAPESP), grant number 2018/23428-0.

## Conflict of Interest

The authors declare that the research was conducted in the absence of any commercial or financial relationships that could be construed as a potential conflict of interest.

## Publisher’s Note

All claims expressed in this article are solely those of the authors and do not necessarily represent those of their affiliated organizations, or those of the publisher, the editors and the reviewers. Any product that may be evaluated in this article, or claim that may be made by its manufacturer, is not guaranteed or endorsed by the publisher.
