# Supplementary material for: Myosteatosis Differentially Affects the Prognosis of Non-Metastatic Colon and Rectal Cancer Patients: An Exploratory Study
Source: Front Oncol. 2021 Nov 11;11:762444. doi: 10.3389/fonc.2021.762444 (PMC8632142; doi:10.3389/fonc.2021.762444)
Supplement: Supplementary file 1 [file DataSheet_1.pdf]

Supplementary Material

1 **Supplementary table 1. Body composition and inflammatory indexes according to myosteatosis of stage I-III colon cancer patients.**

Characteristic	All-patients, n=118	No myosteatosis, n=32	Myosteatosis, n=86	P value
**Skeletal muscle**				
Area (cm^2), mean (SD)				
All Patients	120.8 (31.8)	127.7 (32.8)	118.3 (31.3)	0.155[a]
Female	97.5 (19.6)	100.9 (15.0)	96.2 (21.1)	0.422[a]
Male	141.9 (25.4)	154.5 (21.4)	137.5 (25.5)	**0.020[a]**
Radiodensity (HU), mean (SD)				
All Patients	32.2 (9.3)	43.7 (6.0)	27.8 (6.0)	
Female	31.1 (9.7)	42.7 (6.3)	26.5 (6.4)	
Male	33.1 (8.9)	44.8 (5.6)	29.0 (5.5)	
SMI (cm^2/m^2), mean (SD)				
All Patients	45.4 (9.3)	47.8 (9.3)	44.5 (9.3)	0.101[a]
Female	40.4 (7.3)	41.4 (6.7)	40.1 (7.6)	0.569[a]
Male	49.7 (8.8)	53.8 (7.3)	48.4 (8.9)	**0.037[a]**
IMAT, area (cm^2), median (IQR)				
All Patients	8.4 (5.3–12.8)	4.6 (3.3–5.7)	10.7 (7.0–14.5)	**<0.001[b]**
Female	8.2 (5.1–14.2)	4.0 (2.5–6.2)	11.0 (6.1–17.7)	**<0.001[b]**
Male	8.4 (5.4–12.3)	4.8 (3.9–5.7)	10.3 (7.7–13.2)	**<0.001[b]**
**Visceral adipose tissue**				
VAT area (cm^2), median (IQR)				
All Patients	95.1 (40.6–166.4)	74.5 (33.2–139.5)	101.9 (48.3–171.1)	0.125[b]

Female	81.9 (37.4–130.1)	47.6 (27.9–93.3)	95.1 (42.2–136.0)	0.092[b]
Male	112.1 (51.6–193.6)	95.3 (46.2–160.7)	117.8 (51.6–204.9)	0.499[b]
VATI (cm^2/m^2), median (IQR)				
All Patients	36.3 (16.9–60.4)	28.5 (14.1–48.2)	40.0 (18.6–62.0)	0.170[b]
Female	32.8 (15.6–51.3)	17.5 (13.5–34.5)	39.4 (19.2–57.0)	0.079[b]
Male	40.2 (19.9–71.7)	36.3 (21.6–60.4)	40.5 (18.6–72.9)	0.675[b]
VAT attenuation (HU), median (IQR)				
All Patients	-93.4 (-100.3 to -83.3)	-92.2 (-99.17 to -86.19)	-93.9 (-100.6 to -82.8)	0.846[b]
Female	-93.7 (-101.0 to -83.0)	-92.2 (-101.3 to -81.1)	-94.3 (-99.9 to -83.1)	0.957[b]
Male	-93.1 (-99.5 to -83.3)	-92.5 (-96.7 to -87.8)	-93.1 (-102.2 to -82.8)	0.898[b]
**Subcutaneous adipose tissue**				
SAT area (cm^2), median (IQR)				
All Patients	118.8 (83.3–199.1)	114.2 (94.1–164.7)	128.0 (76.3–199.9)	0.746[b]
Female	178.6 (105.5–256.3)	154.6 (94.1–250.7)	182.0 (116.0–271.0)	0.404[b]
Male	102.5 (54.9–140.6)	108.2 (79.4–117.3)	93.8 (51.2–150.1)	0.640[b]
SATI (cm^2/m^2), median (IQR)				
All Patients	45.9 (31.4–72.9)	39.1 (36.4–57.3)	46.7 (27.9–83.7)	0.708[b]
Female	71.3 (46.4–105.3)	60.7 (38.7–96.8)	72.9 (48.4–113.6)	0.304[b]
Male	36.5 (20.5–46.5)	37.2 (33.0–41.4)	34.0 (18.8–47.0)	0.556[b]
SAT attenuation (HU), median (IQR)				
All Patients	-100.7 (-108.0 to -91.4)	-99.6 (-104.5 to -96.0)	-101.7 (-108.5 to -84.5)	0.753[b]
Female	-104.8 (-112.2 to -94.0)	-102.8 (-113.0 to -96.5)	-105.4 (-112.2 to -92.7)	0.711[b]
Male	-97.4 (-103.7 to -84.5)	-97.6 (-101.4 to -92.4)	-97.1 (-104.8 to -80.2)	0.847[b]

**Inflammatory indexes**				
NLR, median (IQR)	3.1 (2.1–4.8)	2.9 (2.0–3.9)	3.7 (2.1–5.6)	0.211[b]
LMR, median (IQR)	2.7 (1.8–3.8)	3.4 (2.5–4.9)	2.5 (1.7–3.3)	**0.013**[b]
PLR, median (IQR)	163.5 (127.1–265.3)	132.4 (99.3–163.2)	179.8 (133.6–282.3)	**0.003**[b]

Abbreviations: HU: Hounsfield Units; IMAT: Intramuscular adipose tissue; IQR: Interquartile Range; NLR: Neutrophil to Lymphocyte Ratio; LMR: lymphocyte to monocyte ratio; PLR: Platelet to Lymphocyte Ratio; SAT: subcutaneous adipose tissue; SD: Standard Deviation; SATI: Subcutaneous Fat Index; SMI: Skeletal Muscle Index; VAT: Visceral adipose tissue; VATI: Visceral Fat Index. aStudent’s T test; bMann-Whitney test.

Supplementary table 2. Body composition and inflammatory indexes according to myosteatosis of stage I-III rectal cancer patients.

Characteristic	All-patients, n=109	No myosteatosis, n=45	Myosteatosis, n=64	P value
**Skeletal muscle**				
Area (cm^2), mean (SD)				
All Patients	134.3 (30.6)	144.1 (32.8)	127.4 (27.2)	**0.005**[a]
Female	112.5 (23.5)	122.9 (27.4)	105.3 (17.4)	**0.011**[a]
Male	150.2 (25.0)	159.6 (27.5)	143.6 (21.1)	**0.011**[a]
Radiodensity (HU), mean (SD)				
All Patients	34.4 (10.1)	43.3 (7.1)	28.1 (6.6)	
Female	32.6 (9.8)	41.4 (5.5)	26.5 (7.0)	
Male	35.6 (10.3)	44.8 (7.9)	29.2 (6.1)	
SMI (cm^2/m^2), mean (SD)				
All Patients	50.8 (9.3)	54.3 (9.1)	48.3 (8.7)	**0.001**[a]
Female	46.8 (9.5)	51.2 (10.2)	43.8 (7.9)	**0.008**[a]
Male	53.7 (8.0)	56.7 (7.4)	51.6 (7.7)	**0.011**[a]
IMAT, area (cm^2), median (IQR)				
All Patients	9.2 (6.3–15.4)	7.3 (5.3–8.6)	13.4 (8.7–18.3)	**<0.001**[b]
Female	9.8 (7.5–15.9)	8.5 (5.8–9.7)	15.0 (8.6–18.7)	**<0.001**[b]
Male	9.2 (6.0–14.6)	6.4 (4.5–8.0)	13.0 (9.2–17.9)	**<0.001**[b]
**Visceral adipose tissue**				
VAT area (cm^2), median (IQR)				
All Patients	128.9 (74.4–191.3)	101.3 (56.3–163.5)	149.2 (83.7–218.1)	**0.006**[b]

Female	117.2 (65.0–174.3)	94.3 (49.0–151.3)	128.4 (74.4–199.5)	**0.041**[b]
Male	141.9 (78.0–210.0)	122.8 (62.2–174.3)	170.1 (88.2–224.5)	**0.049**[b]
VATI (cm^2/m^2), median (IQR)				
All Patients	48.4 (28.0–71.2)	39.5 (21.9–61.4)	55.4 (32.5–82.9)	**0.004**[b]
Female	46.4 (28.3–74.3)	38.0 (22.7–67.2)	54.2 (31.9–89.4)	**0.039**[b]
Male	48.9 (27.6–70.2)	42.3 (21.0–61.1)	56.5 (33.0–80.3)	**0.032**[b]
VAT attenuation (HU), median (IQR)				
All Patients	-97.2 (-103.7 to -90.3)	-96.18(-100.0 to -85.2)	-101.8 (-105.1 to -93.1)	**0.004**[b]
Female	-98.6 (-103.4 to -93.1)	-96.4 (-100.8 to -91.5)	-102.0 (-103.9 to -93.1)	0.090[b]
Male	-96.8 (-104.8 to -88.6)	-93.2 (-99.8 to -83.9)	-99.5 (-105.9 to -93.2)	**0.017**[b]
**Subcutaneous adipose tissue**				
SAT area (cm^2), median (IQR)				
All Patients	131.6 (89.5–205.6)	117.5 (82.6–210.2	142.9 (93.5–204.7)	0.763[b]
Female	199.7 (145.6–273.6)	211.6 (162.5–273.6)	190.2 (145.1–281.5)	0.189[b]
Male	102.2 (78.3–133.4)	92.2 (78.3–117.5)	108.7 (82.4–153.6)	0.361[b]
SATI (cm^2/m^2), median (IQR)				
All Patients	48.8 (34.8–82.6)	43.8 (32.0–84.4)	52.1 (37.3–78.7)	0.361[b]
Female	85.2 (60.9–112.1)	86.1 (73.2–97.3)	81.2 (59.8–112.5)	0.798[b]
Male	38.4 (29.6–46.7)	34.4 (30.1–42.8)	39.8 (29.6–51.9)	0.141[b]
SAT attenuation (HU), median (IQR)				
All Patients	-102.5 (-108.3 to -96.9)	-99.5 (-104.7 to -93.9)	-104.8 (-109.8 to -97.9)	**0.004**[b]
Female	-106.0 (-111.1 to -102.1)	-104.3 (-107.7 to -100.1)	-107.3 (-113.0 to -104.4)	**0.032**[b]

Male	-98.7 (-105.0 to -93.1)	-97.2 (-100.0 to -91.6)	-100.2 (-108.4 to -96.7)	**0.021**[b]
**Inflammatory indexes**				
NLR, median (IQR)	2.4 (1.7–3.4)	2.4 (1.6–3.4)	2.3 (1.7–3.4)	0.877[b]
LMR, median (IQR)	3.3 (2.4–4.8)	3.4 (2.6–5.0)	3.3 (2.0–4.5)	0.227[b]
PLR, median (IQR)	133.5 (110.6–178.3)	126.3 (106.0–168.5)	139.9 (113.7–190.5)	0.256[b]

Abbreviations: HU: Hounsfield Units; IMAT: Intramuscular adipose tissue; IQR: Interquartile Range; NLR: Neutrophil to Lymphocyte Ratio; LMR: lymphocyte to monocyte ratio; PLR: Platelet to Lymphocyte Ratio; SAT: subcutaneous adipose tissue; SD: Standard Deviation; SATI: Subcutaneous Fat Index; SMI: Skeletal Muscle Index; VAT: Visceral adipose tissue; VATI: Visceral Fat Index. aStudent’s T test; bMann-Whitney test.
